# Risk factors for early neurologic deterioration in single small subcortical infarction without carrier artery stenosis: predictors at the early stage

**DOI:** 10.1186/s12883-023-03128-3

**Published:** 2023-02-27

**Authors:** Di Jin, Jing Yang, Hui Zhu, Yuexia Wu, Haichao Liu, Qi Wang, Xiaoyun Zhang, Yanhua Dong, Bin Luo, Yong Shan, Lvming Zhang, Peifu Wang, Jichen Du

**Affiliations:** 1 Department of Neurology, Aerospace Center Hospital, Yuquan Road 15, Haidian District, Beijing, 100049 People’s Republic of China; 2 Graduate School of the First Hospital of Hebei Medical University, Hebei Medical University, Shijiazhuang, People’s Republic of China

**Keywords:** Single small subcortical infarction, Stroke, Lacunar infarction, Early neurological deterioration, Risk factor

## Abstract

**Objectives:**

This study aimed to assess the epidemiological features and explore the potential risk factors for early neurological deterioration (END) in patients with acute single small subcortical infarction (SSSI) who underwent antiplatelet therapy without carotid artery stenosis.

**Materials & methods:**

Patients with SSSI, as confirmed by cranial magnetic resonance imaging (MRI), who were hospitalized within 48 h after the onset of symptoms were enrolled. END was mainly defined as increment in the National Institutes of Health Stroke Scale (NIHSS) score of ≥ 2 points or any new neurological deficit. Poor functional outcome was defined as modified Rankin Scale (mRS) score of > 2 points at 3-month after the onset. The association of END with multiple indicators was assessed at the early stage of admission using multivariate logistic regression analysis, and adjusted odds ratios (aORs) were calculated.

**Results:**

A total of 280 patients were enrolled from June 2020 to May 2021, of whom, END occurred in 44 (15.7%) patients (median age, 64 years; 70.5% male), while END occurred during sleep in 28 (63.6%) patients. History of hypertension (aOR: 4.82, *p* = 0.001), infarction in internal capsule (aOR: 3.35, *p* = 0.001), and elevated level of low-density lipoprotein cholesterol (LDL-C; aOR: 0.036, *p* = 0.0016) were significantly associated with the risk of END. Patients with END (aOR: 5.74, *p* = 0.002), history of diabetes (aOR: 2.61, *p* = 0.020), and higher NIHSS scores at discharge (per 1-score increase, aOR: 1.29, *p* = 0.026) were associated with the poor functional outcome at 3-month after the onset.

**Conclusion:**

Patients with a history of hypertension, infarction in internal capsule or a higher level of LDL-C were found to be at a higher risk of END.

**Supplementary Information:**

The online version contains supplementary material available at 10.1186/s12883-023-03128-3.

## Introduction

Single small subcortical infarction (SSSI), which also called lacunar stroke, is a common manifestation in acute ischemic stroke, with an incidence of approximately 15–25% in different populations [1]. The pathogenesis of SSSI is heterogeneous. Lipohyalinosis is the most typical cause, while large parent arterial disease is another underlying etiology [2]. The majority of symptoms of SSSI are commonly mild, and the general clinical prognosis is relatively satisfactory in stroke population. However, some patients, despite receiving standard antiplatelet and statin therapy, still develop neurological deterioration, such as limb motor dysfunction, dysphagia, or progressive aggravation of consciousness within 48–72 h after the onset. This progress is difficult to prevent, and some patients may eventually develop to severe, even life-threatening, functional impairment. This phenomenon is called early neurological deterioration (END) [3]. END is generally associated with less favorable outcomes than patients without deterioration [4]. Therefore, it is essential to assess the risk factors and prevention of END.

Studies have shown that subtype of large artery atherosclerosis with severe proximal or intracranial atherosclerosis is an independent risk factor for END with higher odds ratios (ORs) than other subtypes of stroke[5–8]. However, patients with small vessel disease (SVD), i.e., without obvious carotid artery stenosis on radiological imaging, are also at the risk of worsening symptoms in the acute phase of cerebral infarction [4, 9]. Because of the relatively mild symptoms at the onset and the negative findings on vascular imaging, this group of patients may miss the optimal period of treatment, which may lead to the poor prognosis. The pathogenesis and risk factors of END have still remained controversial due to the lack of reliable evidence [10]. Therefore, predicting the risk of END through clinical manifestations, laboratory tests, and radiological results in the early stage of the stroke has noticeably attracted scholars’ attention in recent years. The present study aimed to evaluate the epidemiological features, explore the potential risk factors for END in patients with SSSI as confirmed by cranial magnetic resonance imaging (MRI), and provide evidence for the clinical practice at the early stage of the stroke.

## Materials & methods

### Patients’ selection

This single-center, imaging-based, cross-sectional cohort study with 3-month of clinical follow-up included SSSI patients who were consecutively admitted to the Aerospace Center Hospital in Beijing (China) between June 2020 and May 2021. If there were no contraindications, all patients were routinely treated with aspirin and/or clopidogrel for antiplatelet aggregation and statin for intensive lipid-lowering. Patients with SSSI confirmed by cranial magnetic resonance diffusion-weighted imaging (MR-DWI) and apparent diffusion coefficient (ADC) were included, and the lesion needed to be the main cause of the stroke. Subcortical infarction was diagnosed as a small infarct in the territory of perforating arteriole with maximum diameter of less than 20 mm in the axial plane [11], while without limitation of layers of axial plane. All enrolled patients were hospitalized within 48 h after the onset of symptoms. Patients with a history of ischemic stroke could be included, while they needed to meet the modified Rankin Scale (mRS) score of 0–2. Patients with moderate-to-severe carotid artery stenosis (≥ 50%) or chronic total occlusion of the adjacent major coronary arteries, multiple lesions or watershed cerebral infarcts, cortical infarction, cardioembolism, stroke mimics, or MR-negative strokes were excluded. Patients with other diseases that might aggravate the condition, such as severe pneumonia, septic shock, or severe cardiac insufficiency, were also excluded.

### Collection of clinical data

The following baseline characteristics were collected: (1) Demographic variables: age, gender, and mRS score before onset; (2) Medical history: hypertension (previous antihypertensive medication usage, systolic blood pressure (SBP) ≥ 140 mmHg, or diastolic blood pressure (DBP) ≥ 90 mmHg at discharge), diabetes (previous use of medication or hemoglobin A1c > 7.0%), dyslipidemia (previous usage of lipid-lowering medication, low-density lipoprotein cholesterol (LDL-C) > 3.12 mmol/L, total cholesterol (TC) > 5.17 mmol/L, or triglycerides (TG) > 1.7 mmol/L), previous ischemic stroke, and habitual smoking (current or past regular smoking); (3) Clinical features: National Institutes of Health Stroke Scale (NIHSS) score on admission, SBP and DBP on admission, subtypes of lacunar syndrome, time interval between the onset of symptoms and time of admission, undergoing thrombolysis using recombinant tissue-type plasminogen activator (rt-PA); (4) Laboratory data on admission: leukocyte count, TC, LDL-C, blood glucose, uric acid, blood urea nitrogen (BUN), creatinine (CR), and D-dimer.

### Definition of END and poor functional outcome at 3-month

END was defined as any new neurological symptoms or worsening that might occur within 48 h after the onset of the stroke and persist for at least 24 h. Specifically, END should meet the at least one of the following criteria: (1) An increment in the total NIHSS score of ≥ 2 points, (2) An increment in the consciousness score (1a-1c) of ≥ 1 point, (3) An increment in the motor score (5a-6b) of ≥ 1 point, or (4) Any new neurological deficits that would be unmeasurable by NIHSS scores [9]. The NIHSS scores of all participants were evaluated by neurologists every 6 h at the first 48 h of hospitalization and at least once a day thereafter. When the patient had END symptoms, doctors on duty evaluated the NIHSS score at the first time, recorded the time from the onset to END, and performed the cranial computed tomography to exclude intracranial hemorrhage. In addition, the poor functional outcome was defined as mRS score of 3–6 points at 3-month after the onset by telephone or face-to-face consultation.

### Assessment of neuroimaging data

All participations underwent MRI on a 1.5 or 3.0 Tesla scanner (1.5 Tesla MAGNETOM Avanto; 3.0 Tesla MAGNETOM Skyra; Siemens, Erlangen, Germany) within 24 h after admission. Moreover, DWI, ADC, fluid-attenuated inversion recovery, and time-of-flight MR angiography were conducted according to the routine protocol of stroke. Two experienced vascular neurologists, who were blinded to the clinical data, reviewed all imaging data, and selected eligible participants (κ-value, 0.89). Radiological features (location of the infarction, branch atheromatous disease [BAD], and visible layers of axial slices on DWI) were recorded. BAD of the lenticulostriate arteries was defined as infarcts with the maximum diameter of 10–20 mm on axial slices and being visible for no less than three axial slices, and that of the anterior pontine arteries was defined as unilateral infarcts extending to the basal surface of the pons [12].

### Statistical analysis

Data were presented as mean with standard deviation, median with interquartile range (IQR), and percentage for continuous, ordinal, and categorical variables, respectively. To compare the baseline data between the two groups, the Student’s *t*-test was used for normally distributed data, as well as the Wilcoxon rank-sum test or the Kruskal–Wallis test for abnormally distributed continuous and ordinal variables, and the Pearson's χ^2^, the Fisher's exact test or the Cochran-Mantel–Haenszel χ^2^ test for categorical variables. The Cohen κ coefficient was used to measure inter-rater reliability for qualitative (categorical) items.

A binomial logistic regression model was utilized to assess the association between variables and END. The variables imported into the univariate regression analysis were obtained from characteristics with between-group differences in baseline data (*P* ≤ 0.1) and the probable risk factors of END that were confirmed in previous studies [age, gender, location in corona radiata, infarction in internal capsule and brainstem [4, 13, 14]; BAD [12]; visible layers on DWI [15]; history of diabetes [16]; blood pressure on admission [17]; leukocyte count [18]; glucose [19]; hypertriglyceridemia [20]; D-dimer and uric acid [21]; BUN/CR ratio [22] and D-dimer [23]. A multivariate logistic regression model was used to analyze possible independent factors for END and poor function outcome at 3-month after the onset using variables with *P* ≤ 0.1 in the univariate analysis. The corresponding estimates for ORs with 95% confidence intervals (CIs) were presented. We use area under the receiver operating characteristic (ROC) curve to evaluate the validation of the model.

Moreover, EpiData 3.0 software was used to collect data and establish the database. The statistical analysis was conducted using R 4.2.0 software. Two-sided *P* < 0.05 was considered statistically significant.

## Results

**Fig. 1:**
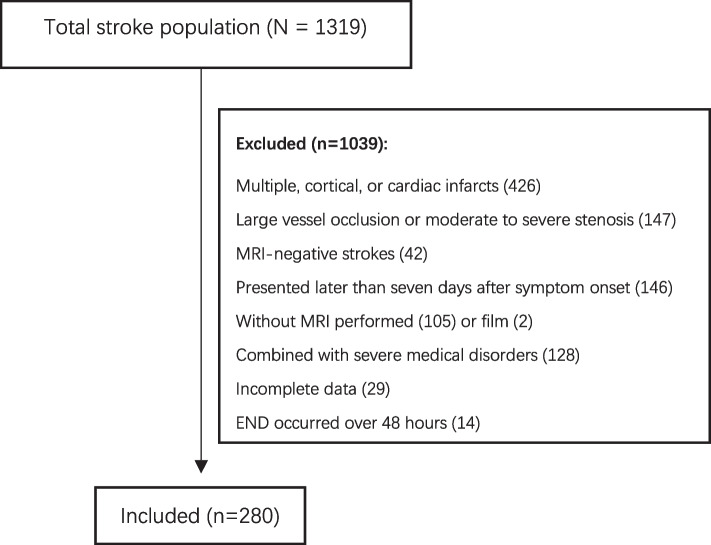
The Selection process of the study

Of 1,319 cases with SSSI, 280 (21.2%) cases were included in the final analysis. Figure 1 shows patients’ selection process. Men comprised 70.7% (*n =* 198) of the total, and the median age was 65 (IQR, 57–73) years. Median NIHSS scores were 2 (IQR, 1–3) points on admission and 1 (IQR, 0–2) point at discharge. A total of 44 (15.7%) patients progressed to END within 48 h after the onset, while 236 (84.3%) patients were clinically stable. In the END group, 20 (45.5%) and 24 (54.5%) patients met the diagnostic criteria for END on the first and the second days after the onset of symptoms, respectively. No END patient developed with intracranial hemorrhage or death during hospitalization. Furthermore, END occurred in 28 (63.6%) patients during sleep, and END occurred in 16 (36.4%) patients during wakefulness or activity. In addition, 43.2% (19/44) of patients were deteriorated prior to admission, while 56.8% (25/44) of patients were exacerbated during hospitalization. Comparison of the NIHSS scores of the pre-admission END and after-admission END revealed that there was no statistically significant difference between the two subgroups (*P* = 0.36). At the peak of the disease course, the median NIHSS score of END patients was elevated by 6 (IQR, 4–8) points.

The baseline data of the END group and the clinically stable group were basically similar in the majority of features. There were some differences between the two groups. Patients with END were more likely to have history of hypertension (*P* = 0.033) and infarction in internal capsule (*P* = 0.003). Besides, patients in the END group had slightly higher NIHSS scores on admission (*P* = 0.060), slightly higher levels of LDL-C (*p* = 0.063), and more visible layers of axial slices on DWI (*P* = 0.061). Details of baseline characteristics in different groups are presented in Table 1.

**Table 1:** Baseline characteristics of clinically stable group, END group and in total

	Clinically Stable(*n =* 236)	END (*n =* 44)	Total (*n =* 280)	*p* value
**Demographics**				
Age, y	66 (57–74)	64 (55–70)	65 (57–73)	0.084
Male, n (%)	167 (70.8)	31 (70.5)	198 (70.7)	0.96
Pre-stroke modified Rankin Scale score	0 (0–0)	0 (0–0)	0 (0–0)	0.14
**Medical history, n (%)**				
Hypertension	144 (61.0)	37 (84.1)	181 (64.6)	*0.033*
Diabetes	89 (37.7)	18 (40.9)	107 (38.2)	0.69
Dyslipidemia	57 (24.2)	9 (20.5)	66 (23.6)	0.56
Previous stroke	61 (25.8)	9 (20.5)	70 (25.0)	0.45
Smoke	85 (36.0)	17 (38.6)	102 (36.4)	0.74
**Clinical features**				
NIH Stroke Scale score on admission	2 (1–3)	2 (1–3)	2 (1–3)	0.060
Time from onset to admission, day	2 (1–3)	2 (1–2)	2 (1–3)	0.16
Systolic blood pressure, mmHg	145 (133–160)	144 (132–156)	145 (132–159)	0.86
Diastolic blood pressure, mmHg	80 (74–93)	82 (78–92)	81 (74–92)	0.49
Subtypes of lacunar syndrome, n (%)				
Pure motor stroke	140 (59.3)	25 (56.8)	165 (58.9)	0.76
Ataxic hemiparesis	28 (11.9)	8 (18.2)	36 (12.9)	0.25
Sensorimotor stroke	43 (18.2)	11 (25.0)	54 (19.3)	0.40
Pure sensory stroke	19 (8.1)	0 (0)	19 (6.8)	0.051
Atypical lacunar syndrome	6 (2.5)	0 (0)	6 (2.1)	0.59
Thrombolysis treatment (rt-PA), n (%)	14 (5.9)	6 (13.6)	20 (7.1)	0.068
**Magnetic resonance imaging features**				
Left, n (%)	134 (56.8)	28 (63.6)	162 (57.9)	0.65
Visible layers of axial slices				0.061
Layers = 1	97 (41.1)	11 (25.0)	108 (38.6)	
Layers = 2	81 (34.3)	21 (47.7)	102 (36.4)	
Layers = 3	43 (18.2)	6 (13.6)	49 (17.5)	
Layers ≥ 4	15 (6.4)	6 (13.6)	21 (7.5)	
Branch atheromatous disease, n (%)	58 (24.6)	17 (38.6)	75 (26.8)	0.17
Lesion location				
Thalamus, n (%)	39 (16.5)	3 (6.8)	42 (15.0)	0.10
Corona radiata, n (%)	62 (26.3)	9 (20.5)	71 (25.4)	0.42
Internal capsule, n (%)	57 (24.2)	20 (45.5)	77 (27.5)	*0.003*
Brainstem, n (%)	65 (27.5)	12 (27.3)	77 (27.5)	0.97
Subcortical, n (%)	13 (5.5)	0 (0)	13 (4.6)	0.23
**Laboratory values (on admission)**				
White blood cell, 10^9/L	6.69 (5.59–7.95)	6.72 (5.97–8.23)	6.69 (5.69–8.05)	0.41
Neutrophils, 10^9/L	4.43 (3.55–5.56)	4.61 (3.73–5.97)	4.50 (3.55–5.59)	0.42
LDL cholesterol, mmol/L	2.65 (2.11–3.18)	3.01 (2.32–3.54)	2.68 (2.12–3.22)	0.063
Total cholesterol, mmol/L	4.58 (3.80–5.18)	4.88 (4.28–5.58)	4.60 (3.87–5.31)	0.11
Triglycerides, mmol/L	1.35 (0.98–1.98)	1.42 (1.02–2.12)	1.36 (0.99–1.98)	0.64
Glucose, mmol/L	6.54 (5.48–9.39)	7.53 (5.88–11.29)	6.66 (5.53–9.55)	0.15
Uric acid, μmol/L	334.00 (277.82–385.18)	314.25 (276.72–364.08)	330.35 (277.22–383.10)	0.22
BUN, mg/dL	9.90 (7.92–11.52)	9.63 (7.70–11.52)	9.90 (7.88–14.44)	0.74
Cr, mg/dl	0.82 (0.69–0.97)	0.77 (0.64–0.90)	0.81 (0.67–0.95)	0.12
BUN/ Cr	12.35 (8.68–16.02)	13.09 (10.36–14.57)	11.94 (9.72–14.44)	0.19
D-dimer, ug/L	106(70–205)	110 (76–146)	106(71–196)	0.99
**Evaluation at Discharge**				
NIH Stroke Scale score	0 (0–2)	4 (2–6)	1 (0–2)	< 0.001
Modified Rankin Scale score	0 (0–1)	2 (0–4)	0 (0–2)	< 0.001

*END* Early neurologic deterioration, *rt-PA* Recombinant tissue-type plasminogen activator, *LDL* Low-density lipoprotein, *BUN* Blood urea nitrogen, *Cr* Creatinine

The results of univariate and multivariate logistic regression models related to the predictors of END are listed in Table 2. Multivariate logistic regression models adjusted for relevant confounders showed that history of hypertension (adjusted OR (aOR): 4.82 [95%CI: 1.95–11.96], *P* = 0.001), infarction in internal capsule (aOR: 3.35 [95%CI: 1.64–6.83], *P* = 0.001), and the highest quartile of LDL-C (aOR: 3.30 [95%CI: 1.25–8.70], *P* = 0.016) were identified as independent predictors of END. In contrast, age, BAD, visible layers of axial slices on DWI, and other quartiles of LDL-C were not significantly associated with END. The area under ROC curve of the model is 0.735 [95%CI, 0.680–0.786, *p* < 0.0001], with sensitivity of 56.8% and specificity of 79.7%.

**Table 2:** Results of logistic regression analysis for predictors of END

Variables	Univariate Models		Multivariable Models	
	Crude OR [95% CIs]	*p* value	Adjusted OR [95% CIs]	*p* value
Age	0.98 [0.95–1.00]	0.068	0.99 [0.96–1.02]	0.35
History of hypertension	3.38 [1.45–7.89]	0.005	4.83 [1.95–11.96]	0.001
NIHSS score on admission	1.08 [0.93–1.26]	0.31	1.08 [0.91–1.28]	0.39
BAD	1.80 [0.93–3.47]	0.080	1.58 [0.76–3.28]	0.23
Visible Layers of axis slices	1.31 [0.99–1.73]	0.054	1.07 [0.65–1.75]	0.79
Infarction in internal capsule	2.62 [1.35–5.08]	0.005	3.35 [1.64–6.83]	0.001
LDL-C quartile	Ref		Ref	
LDL-C quartile (1)	1.38 [0.51–3.71]	0.53	1.51 [0.54–4.24]	0.43
LDL-C quartile (2)	1.35 [0.50–3.65]	0.55	1.52 [0.54–4.27]	0.42
LDL-C quartile (3)	2.69 [1.06–6.80]	0.036	3.30 [1.25–8.70]	0.016

LDL-C quartile (ref.): 0.92–2.15 mmol/L, LDL-C quartile (1): 2.16–2.69 mmol/L, LDL-C quartile (2): 2.70–3.24 mmol/L, LDL-C quartile (3): ≥ 3.25 mmol/L. Effects are presented as adjusted odds ratios with 95% CI. The pseudo-R^2^ of the model is 0.092

*END* Early neurologic deterioration, *OR* Odds ratio, *Cis* Confidence intervals, *BAD* Branch atheromatous disease, *LDL-C* Low-density lipoprotein cholesterol

**Fig. 2:**
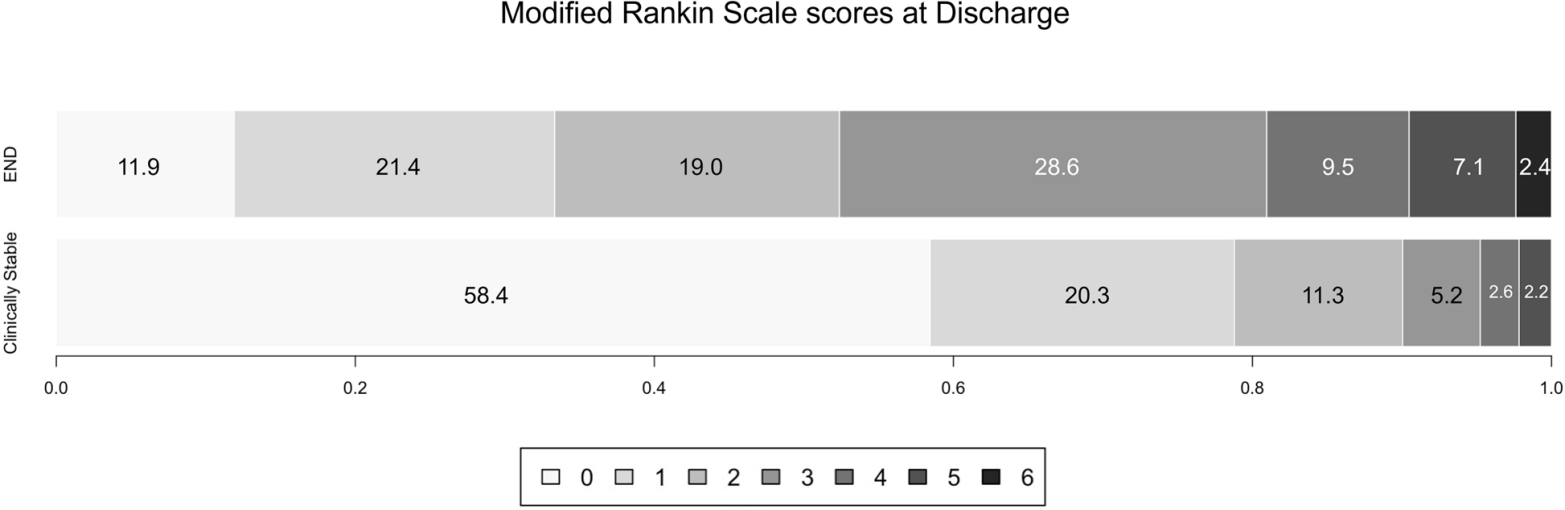
Comparison of clinical outcome (modified Rankin scale scores at 3-month from onset) between early neurological deterioration group and clinically stable group

Furthermore, 3-month mRS scores were obtained for 273 (97.5%) of 280 patients. 15.8% (43/273) of patients had an mRS score ≥ 3, with the incidence in the END group (20/42, 47.6%) was significantly higher than the clinical stable group (9.96%, 23/231). Patients in the END group had more severe neurological deficits with higher NIHSS scores and mRS scores (*P* < 0.001, Table 1 and Fig. 2). In the multivariate logistic regression analysis of predictors of poor function (Table 3), after adjustment for confounders, END (aOR: 5.74 [95%CI: 1.89–17.45], *P* = 0.002), history of diabetes (aOR: 2.61 [95%CI: 1.16–5.84], *P* = 0.020), and higher NIHSS scores at discharge (per 1-score increase, aOR: 1.29 [95%CI: 1.03–1.61], *P* = 0.026) were associated with a less favorable functional outcome at 3-month after the onset. The area under ROC curve of the model is 0.865 [95%CI, 0.819–0.903, *p* < 0.0001], with sensitivity of 81.4% and specificity of 80.9%.

**Table 3:** Results of logistic regression analysis for predictors of poor function at 3-month

Variables	Univariate Models		Multivariable Models	
	Crude OR [95% CI]	*p* value	Adjusted OR [95% CI]	*p* value
END	8.22 [3.90–17.29]	< 0.001	5.74 [1.89–17.45]	0.002
Age (per 1 year)	1.03 [0.99–1.05]	0.064	1.03 [0.99–1.07]	0.071
History of diabetes	2.66 [1.37–5.16]	0.005	2.61 [1.16–5.84]	0.020
NIHSS score on admission	1.47 [1.25–1.73]	< 0.001	1.24 [0.96–1.60]	0.094
Visible layers of axis slices	1.29 [0.97–1.70]	0.077	1.03 [0.71–1.49]	0.88
NIHSS score at discharge	1.64 [1.40–1.92]	< 0.001	1.29 [1.03–1.61]	0.026

*OR* Odds ratio, *Cis* Confidence intervals, *END* Early neurologic deterioration, *NIHSS* NIH stroke scale

Effects are presented as adjusted odds ratios with 95% CI. The pseudo-R^2^ of the model is 0.232

## Discussion

The present study evaluated the incidence and risk factors of END in a cohort of SSSI patients. The main findings were summarized as follows: (1) END was commonly found in SSSI patients without carotid artery stenosis and occurred in 15.7% of patients within 48 h after the onset of symptoms; (2) About two-thirds of patients experienced worsening of symptoms during sleep; (3) Patients with history of hypertension, infarction in internal capsule, and elevated LDL-C level were at the higher risk of END; (4) END, history of diabetes, and higher NIHSS scores at discharge were associated with poor functional outcome at 3-month after the onset.

The incidence of END was about 11–34% in previously reported results [4, 13, 14, 20, 24–27]. However, these studies have differences in the inclusion criteria for END, and some of them did not exclude patients with carotid arteries stenosis or verified the lesion by MRI. To ensure the homogeneity of the study subjects, patients with cranial MRI findings were included, and patients with moderate-to-severe carotid artery stenosis, cardiogenic embolism, and stroke due to other etiologies were excluded. The results indicated that about one in six patients developed END, which was in line with the findings of another MR-based study [4] and indicated that END was not rare in patients with SSSI.

To date, several studies have concentrated on the effects of hypertension on END during the acute phase of cerebral infarction, while their results were inconsistent. Yamamoto et al. found that history of hypertension was an independent factor of END in patients with SVD [8]. He et al. demonstrated that mean SBP within 24 h was the best predictor for END patients who received thrombolysis using rt-PA [28]. Vynckier et al. reported that END patients had mainly history of hypertension slightly, while neither SBP nor mean arterial blood pressure on admission was significantly associated with the risk of END [4]. The present study suggested that the history of hypertension, rather than hypertension on admission, was the risk factor for END. Hypertension is one of the most important risk factors for stroke. In INTERSTROKE study, stroke in 54% of patients was attributed to the history of hypertension or blood pressure higher than 160/90 mmHg [29]. The increase of blood pressure is associated with the increased arterial stiffness, affecting cerebral hemodynamics with microvascular rupture [30]. In SVD, abnormal cerebral pulsatile hemodynamics may cause structural changes and affect small arteries, arterioles, capillaries, and venules, which are finally presented as white matter hyperintensity, microbleeds, brain atrophy, and infarcts on MRI [11, 31]. Therefore, stroke patients with history of hypertension may further progress to END. On the other hand, up to 80% of patients with acute ischemic stroke might experience acute hypertensive response within the first 24 h after the onset [32] and fall back at 4–10 days spontaneously. As a general symptom, the increased blood pressure on admission may transiently fluctuate, and patients’ progression to END mainly depends on the cerebrovascular reserve capacity and the occurrence of secondary side effects (e.g., cerebral edema, hyperfusion, and hemorrhagic transformation) during fluctuation of blood pressure [33].

The location of cerebral infarction may have a predictive value for END. Berberich et al. demonstrated that infarction in the internal capsule or basal ganglia increased the risk of END [13]. Patients with infarcts in the ventral pontine were also at the high risk of END [4, 14]. The present study showed that infarction in internal capsule could increase the risk of progression to END compared with the absence of infarction in this area. This phenomenon could be explained by the higher density of corticospinal tracts in this area, indicating that minor extension of arteriosclerotic plagues may lead to the noticeable progression of symptoms [3]. In addition, infarction in this area may be caused by BAD or hypoperfusion, which are also considered as possible risk factors for END. BAD lesions of lenticulostriate arteries mainly have more than three layers of axial slices on DWI, and those of the anterior pontine arteries are typically characterized by unilateral infarcts extending to the basal surface of the basal pons [12]. The layers of slices were defined as a variable because multiple layers represented the enlargement of the infarct volume, leading to neurological deterioration in patients with SVD [34]. Previous studies indicated that the number of slices significantly differed between patients with and without END [15, 35]. However, in the present study, neither BAD nor the visible layers of axial slices on DWI would be associated with the risk of END. This result indicates that simply considering the location, size or volume of infarcts may be one-sided, and combination with other imaging methods, such as cerebral perfusion and diffusion tensor imaging, may be more predictive [24].

Some scholars have demonstrated that dyslipidemia may be one of the risk factors for END. TC, LDL-C, and TG are the commonly reported lipid metabolic indicators [36]. In the present study, the highest quartile of LDL-C was found to be associated with the increased risk of END. LDL-C plays an important role, as a pro-inflammatory mediator, in the oxidative processes, and patients with a higher LDL-C level may be accompanied with a strong oxidative reaction, leading to the expansion of infarct volume [37]. However, the correlation between serum lipids and END has still remained controversial. A meta-analysis of 2 studies with involvement of 867 patients showed that the elevated level of TG was correlated with the risk of END in patients with acute cerebral infarction, while neither LDL-C nor high-density lipoprotein cholesterol (HDL-C) had a significant prognostic value [38]. These results suggested that the effects of serum lipids on the risk of END should be further assessed.

In the present study, 67.2% of patients experienced worsening of symptoms during sleep, which could be related to the occurrence of nocturnal oxygen desaturation (NOD, defined as pulse oximetry saturation (SpO_2_) < 90%). Kim et al. reported that NOD was an independent risk factor for END and it might mainly occur during nighttime [39]. Nocturnal desaturation may decrease cerebral perfusion and cause compensatory blood pressure surge [40]. Yoon et al. demonstrated that sleep apnea was commonly found in the acute phase of ischemic stroke, and the prevalence of END increased with the level of sleep apnea [41]. These studies have shown that in patients with acute cerebral infarction, assessment of the sleep apnea and nocturnal hypoxia at the early stage of hospitalization may be advantageous to predict the occurrence of END.

In our study, patients with END, history of diabetes and NIHSS score at discharge are associated with poor functional outcome at 3-month follow-up. Patients suffered from END during the stroke course would have 8.22-fold increased risk of poor functional outcome, which is consistent with previous studies [4, 25, 42–44]. Diabetes is confirmed as an independent risk factor for ischemic stroke and may be associated with poor outcomes in previous studies [45, 46]. In our cohort, patients with a history of diabetes were 2.6 times more likely to develop END than those without diabetes. Diabetes influences prognosis of SSSI by several mechanisms, such as involving to the process of chronic inflammatory, atherosclerosis and the formation of plagues [45]. Furthermore, patients with diabetes have an elevated risk of recurrent ischemic event [47].

The present study had some limitations. Firstly, this was a single-center study, and the number of cases in the END group was limited, which might influence subgroup analysis. In the future research, the sample size can be further expanded to obtain more specific and in-depth results. Secondly, as the number of visible layers in the axial slices was considered as a surrogate criterion for judging the size of the lesion, the thickness and number of layers were slightly different for each subject on MRI, and there might be some metrical biases when measuring the infarct volume, which might affect the results. Finally, only routine MRI was used to analyze the radiological features of END patients. In the next research, multimodality imaging methods, e.g., high-resolution MRI of blood vessels, diffusion tensor imaging or cranial perfusion imaging, will be combined to more precisely explore the causes of END.

## Conclusions

Nearly 16% of patients with SSSI experienced END within 48 h after the onset of symptoms. END mainly occurred during sleep in patients. Patients with history of hypertension, infarction in internal capsule, and elevated LDL-C level were at a higher risk of END. END, history of diabetes, and NIHSS score at discharge were found to be associated with the poor functional outcome at 3-month follow-up. Further research is required to evaluate the specific mechanism of END.

## Supplementary Information

**Additional file 1.**

## Data Availability

The datasets analyzed of the logistic regression during the current study are available in the supplementary material. Other baseline datasets are available from the corresponding author on reasonable request.

## Abbreviations

ADC: Apparent diffusion coefficient
BAD: Branch atheromatous disease
CIs: Confidence intervals
BUN: Blood urea nitrogen
Cr: Creatinine
DWI: Diffusion-weighted imaging
END: Early neurological deterioration
HDL-C: High-density lipoprotein cholesterol
IQR: Interquartile ranges
LDL-C: Low-density lipoprotein cholesterol
mRS: Modified Rankin Scale
MR: Magnetic resonance
NIHSS: NIH Stroke Scale
NOD: Nocturnal oxygen desaturation
OR: Odds ratio
rt-PA: Recombinant tissue-type plasminogen activator
SSSI: Single small subcortical infarction
